# Prevalence and Risk Factors of Potentially Malignant Oral Lesion in Prison Population: A Systematic Review

**DOI:** 10.3390/dj14050302

**Published:** 2026-05-14

**Authors:** Erika Roncarati, Saverio Ceraulo, Antonio Barbarisi, Gianluigi Caccianiga, Francesco Carinci, Dorina Lauritano

**Affiliations:** 1 Department of Translational Medicine, University of Ferrara, 44121 Ferrara, Italy; erika01.roncarati@edu.unife.it (E.R.); gianluigi.caccianiga@unife.it (G.C.); crc@unife.it (F.C.); 2 Department of Medicine and Surgery, University of Milano-Bicocca, 20100 Monza, Italy; saverio.ceraulo@unimib.it (S.C.); a.barbarisi@campus.unimib.it (A.B.)

**Keywords:** oral health, prison population, potentially malignant oral disorders, oral lesions, risk factors, incarcerated individuals

## Abstract

**Background:** Potentially malignant oral disorders (OPMDs) and oral carcinomas represent a significant oncological concern in incarcerated populations, where multiple modifiable risk factors such as tobacco use, illicit drug consumption, oncogenic human papillomavirus infections, and poor oral hygiene coexist with limited access to preventive and routine dental care. This combination may increase the risk of delayed diagnosis and malignant transformation. **Objective**: This PRISMA-compliant systematic review aimed to evaluate the prevalence of OPMDs and associated risk factors in prison populations, with a particular focus on identifying gaps in the current evidence. **Methods**. A systematic literature search was conducted in PubMed, Scopus and Cochrane Library using predefined search strategies. The final search yielded 24 records, which were screened according to PRISMA 2020 guidelines. After title and abstract screening, 10 full-text articles were assessed for eligibility, and 5 cross-sectional studies were included in the qualitative synthesis following independent review. **Results:** The included studies revealed a substantial burden of oral mucosal lesions in incarcerated populations. Premalignant lesions were reported in a significant proportion of inmates, with oral submucous fibrosis particularly prevalent in some cohorts. Additionally, a high prevalence of oral high-risk HPV infection and widespread oral manifestations were observed. Tobacco use, often combined with betel quid, alcohol, or illicit drugs, emerged as the primary and consistently associated risk factor for oral lesions. **Conclusions:** Prison populations appear to represent a high-risk group for OPMDs due to the combined effect of behavioral and structural risk factors. However, the limited number of available studies, their cross-sectional design, and methodological heterogeneity prevent definitive conclusions. Further longitudinal and methodologically robust studies are needed to better define prevalence patterns and support targeted screening and prevention strategies in correctional settings.

## 1. Introduction

Oral carcinoma is a major global health burden and ranks among the leading causes of cancer-related morbidity and mortality worldwide. Potentially malignant oral disorders (OPMDs), including leukoplakia, erythroplakia, and oral submucous fibrosis, represent well-established precursors in the multistep process of oral carcinogenesis [1]. Early detection and management of these lesions are essential to reduce malignant transformation and improve clinical outcomes.

Incarcerated populations constitute a high-risk and underserved group, exhibiting a significantly higher prevalence of oral diseases compared to the general population [2,3]. This disparity is primarily attributable to the high burden of behavioral and systemic risk factors, including tobacco use, alcohol consumption, illicit drug abuse, and infections such as human papillomavirus, often compounded by poor oral hygiene, malnutrition, and psychosocial stress [4,5,6,7]. These factors frequently coexist and act synergistically, promoting the onset and progression of oral mucosal alterations.

The spectrum of oral lesions observed in prison populations reflects this complex risk profile. Frequently reported conditions include leukoplakia, erythematous lesions, chronic inflammatory disorders, and persistent mucosal ulcerations [5]. Oral leukoplakia remains the most commonly reported OPMD, strongly associated with tobacco use and alcohol consumption, both of which significantly increase the risk of malignant transformation [5]. Although less prevalent, erythroplakia and mixed red–white lesions are of particular clinical relevance due to their higher malignant potential [8,9].

Geographical variations in the prevalence and distribution of oral lesions have been reported, likely reflecting differences in lifestyle factors, cultural habits, prison healthcare systems, and access to dental care. European studies predominantly report tobacco-related lesions, whereas other regions show a higher prevalence of lesions associated with specific local practices [10,11,12].

The risk of malignant transformation of OPMDs is a critical concern in incarcerated individuals. While the biological mechanisms of oral carcinogenesis are comparable to those in the general population, the prison environment may exacerbate disease progression through persistent exposure to multiple risk factors, chronic mucosal irritation, and impaired tissue repair [13,14]. Additional contributors include untreated dental diseases and drug-induced conditions such as xerostomia, which further compromise oral mucosal integrity [4,7].

Delayed diagnosis represents a major challenge in this setting. The lack of structured screening programs and limited access to routine dental care, often restricted to emergency interventions, may result in prolonged undetected lesions, increasing the likelihood of progression to invasive carcinoma and negatively impacting prognosis [3].

Despite increasing evidence of a high burden of oral disease among prisoners, data on the prevalence and associated risk factors of OPMDs in this population remain fragmented. Therefore, this systematic review aims to synthesize the available evidence on the prevalence of potentially malignant oral lesions and their associated risk factors in prison populations, in order to support targeted preventive strategies and improve early detection in this vulnerable group.

### 1.1. Objectives

The primary objective of this systematic review was to assess the prevalence of potentially malignant oral disorders (OPMDs) in prison populations.

The secondary objective was to identify and analyze the main risk factors associated with the development of OPMDs in incarcerated individuals, with particular focus on behavioral, clinical, and systemic determinants.

### 1.2. Clinical Question (PEO)

Given the observational nature of the included studies, a PEO framework was adopted.

Population (P): incarcerated individuals (prison population).

Exposure (E): behavioral and systemic risk factors (tobacco use, alcohol consumption, illicit drug use, human papillomavirus infection, poor oral hygiene, chronic inflammation).

Outcome (O): prevalence and characteristics of potentially malignant oral disorders (OPMDs).

Due to the observational nature of the included studies and the absence of a defined intervention or comparison group, a PEO framework was adopted instead of PICO.

## 2. Materials and Methods

The PRISMA statement was used to select methods and inclusion criteria, since it provides a reliable protocol for systematic reviews [15].

The review protocol was registered in PROSPERO (CRD420261354600). This systematic review was conducted in accordance with the PRISMA 2020 guidelines, and the corresponding checklist (Table S1) is provided as Supplementary Material.

### 2.1. Eligibility Criteria

#### 2.1.1. Inclusion and Exclusion Criteria

Studies were considered eligible if they met the following inclusion criteria: (1) original observational studies; (2) conducted in incarcerated populations or prison inmates; (3) reporting data on oral mucosal lesions, potentially malignant oral disorders (OPMDs), oral precancerous lesions, or oral conditions from which relevant information on potentially malignant lesions could be extracted; and (4) published in English.

Studies were excluded if they met one or more of the following criteria: (1) reviews, systematic reviews, meta-analyses, editorials, letters, conference abstracts without sufficient data, or case reports; (2) studies not involving prison populations; (3) studies focused exclusively on general dental outcomes, such as caries or periodontal disease, without reporting oral mucosal lesions or potentially malignant conditions; (4) studies lacking sufficient clinical or methodological information; and (5) duplicate records.

Studies were grouped qualitatively according to predominant risk factor assessed (tobacco/betel, illicit drugs, HPV, chronic inflammation).

Because of the limited number of studies specifically addressing OPMDs in prison populations, studies investigating broader oral mucosal lesions were also considered, provided that clinically relevant data related to potentially malignant lesions could be identified and extracted.

#### 2.1.2. Search

A comprehensive literature search was conducted in PubMed, Scopus, and the Cochrane Library. The search strategy was adapted for each database.

Predefined filters were applied to refine the search, including article type (observational studies), publication date (2012–2026), language (English) and human subject.

Data extraction was performed independently by two reviewers (E.R., D.L.) using a standardized form; discrepancies were resolved by discussion.

Searches in PubMed were carried out combining Medical Subject Headings (MeSH) terms with free-text keywords in English, connected by Boolean operators (AND, OR, NOT) and, when appropriate, using parentheses and truncation to optimize sensitivity and specificity; the following search string was used: (“oral lesions” OR “oral mucosal lesions” OR leukoplakia) AND (prisoners OR inmates OR “prison population”).

In Scopus, the search was performed using the TITLE-ABS-KEY function: TITLE-ABS-KEY ((“oral lesions” OR “oral mucosal lesions” OR leukoplakia OR erythroplakia) AND (prisoners OR inmates OR “prison population”)).

In the Cochrane Library, a simplified keyword-based search was conducted using the terms (“oral lesions” AND prisoners). The search strategy was iteratively refined to achieve an optimal balance between sensitivity and specificity.

**Figure 1:**
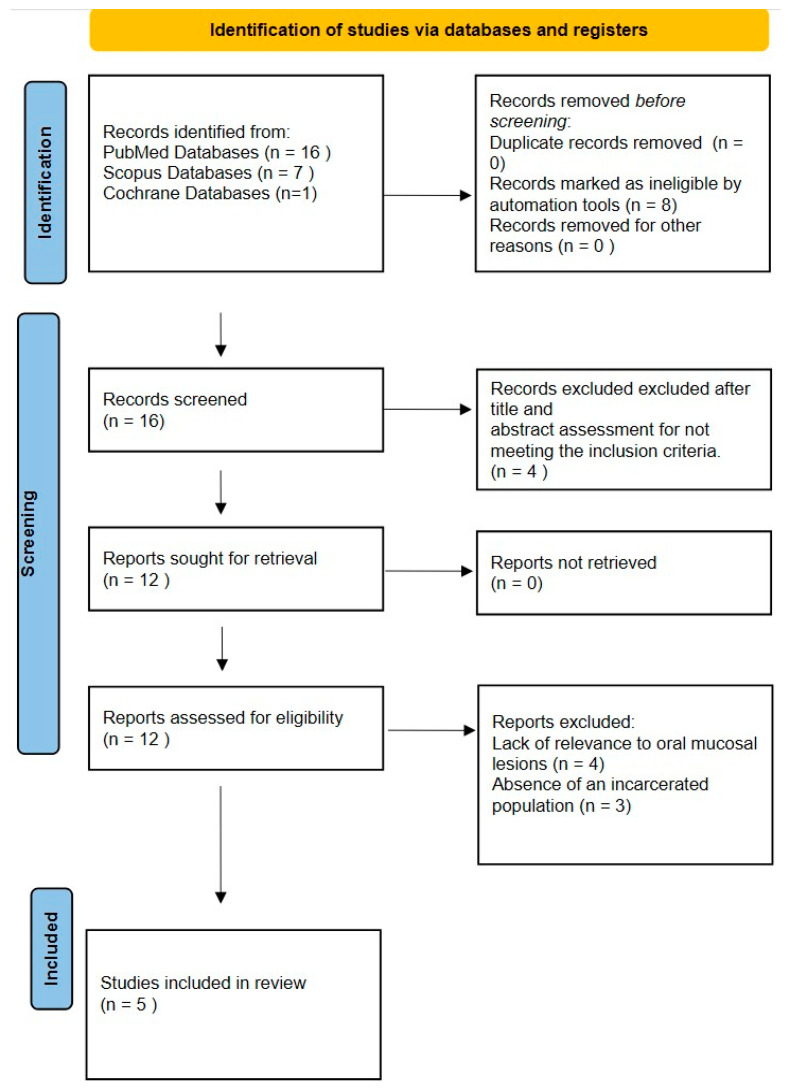
PRISMA flow diagram 2020.

The flow chart that was used for this study is shown in Figure 1.

#### 2.1.3. Outcomes and Data Items

The primary outcomes of interest were measures directly related to malignant and potentially malignant transformation of the oral mucosa in incarcerated adults. Specifically, we sought quantitative data on:

- Oral squamous cell carcinoma and other malignant oral lesions, when reported (incidence or prevalence in prison populations).
- Precancerous and potentially malignant oral lesions, including leukoplakia, oral submucous fibrosis (OSMF), nicotinic palatal keratosis/leukokeratosis, and histologically or clinically defined oral epithelial dysplasia.
- Oral infection with high-risk human papillomavirus (HPV) genotypes (e.g., HPV-16, HPV-59 and other oncogenic types) when associated with dysplastic or potentially malignant oral lesions.

The secondary outcomes were indicators of overall oral disease burden that may contribute to carcinogenesis, namely:

- Dental caries experience, expressed as DMFT (Decayed, Missing and Filled Teeth) indices or equivalent summary measures.
- Periodontal status, assessed by the Community Periodontal Index (CPI) or other validated periodontal indices, and the prevalence of gingivitis and periodontitis.

For each included study, we additionally extracted country and prison setting, study design, sample size, sex distribution, mean age or age range, main oral risk factors assessed (tobacco smoking, smokeless tobacco and betel quid, alcohol, illicit drug use, systemic diseases and medications), indicators of access to dental care, and any reported comparison with the general population or non-exposed groups. When information was missing or unclear, we relied on the published report and did not impute unreported outcomes.

#### 2.1.4. Synthesis Methods

Given the heterogeneity of study designs, populations, settings, and outcome measures, we did not perform a formal meta-analysis. Instead, data were synthesized narratively.

For each included study, we extracted and reported crude prevalence estimates (percentages) for malignant and potentially malignant oral lesions (primary outcomes), and for caries experience (DMFT) and periodontal status (secondary outcomes), when available. Studies were grouped descriptively according to the type of oral lesion/outcome reported (malignant lesions, precancerous/potentially malignant lesions, caries, periodontal disease, HPV infection), and the predominant risk factor profile of the prison population (e.g., tobacco/betel use, illicit drug use, high-risk HPV infection, chronic inflammatory burden and limited access to care).

Where possible, we compared reported prevalences with reference data from the general population cited in the primary studies, to highlight relative excess risk in incarcerated groups. No data transformation, imputation of missing values, or calculation of pooled effect estimates (e.g., risk ratios, odds ratios) was undertaken. Results of individual studies are presented in structured tables and summarized in the text to identify consistent patterns of association between prison-related risk factors and the occurrence of malignant and precancerous oral lesions.

#### 2.1.5. Quality Assessment

The methodological quality of the included studies was assessed using the Newcastle–Ottawa Scale (NOS), adapted for cross-sectional studies. The scale evaluates three domains: selection, comparability, and outcome, with a maximum score of 9 points.

Risk of bias/quality was assessed independently by two reviewers (E.R., D.L.) using the Newcastle–Ottawa Scale for observational studies; disagreements were resolved by consensus.

A total of five studies were assessed, including Relvas [9], Rawlani [6]., Arjun [16], Chaudhari [17]. and Zonta [18]. Overall, the methodological quality was moderate, with NOS scores ranging from 4 to 6.

In the selection domain, most studies achieved 2–3 points due to clearly defined prison populations and appropriate inclusion criteria. However, some studies lacked detailed descriptions of sampling strategies, limiting representativeness.

In the comparability domain, scores were generally low (0–1 point), as few studies adjusted for major confounding factors such as tobacco use, alcohol consumption, or illicit drug use.

In the outcome domain, most studies scored 2 points, as oral conditions were assessed through clinical examination, although standardized diagnostic criteria were not consistently applied.

Overall, the moderate NOS scores indicate a fair level of methodological reliability. However, the cross-sectional design and limited control of confounding factors reduce the strength of the evidence, and findings should therefore be interpreted with caution.

## 3. Results

### 3.1. Study Selection and Characteristics

A total of 24 records were identified through database searching, including 16 from PubMed, 7 from Scopus, and 1 from the Cochrane Library.

All records retrieved from Scopus (n = 7) were excluded during the title and abstract screening phase, as they were not relevant to the research question. Specifically, these articles belonged to non-clinical fields or consisted of conference abstracts, book chapters, and other publication types not meeting the eligibility criteria.

The single record identified through the Cochrane Library (n = 1) was excluded during the screening phase, as it was not pertinent to oral mucosal lesions or potentially malignant disorders in incarcerated populations based on title and abstract evaluation.

The remaining 16 records identified through PubMed were screened, of which 4 were excluded after title and abstract assessment for not meeting the inclusion criteria. Twelve full-text articles were assessed for eligibility.

Of these, 7 studies were excluded due to lack of relevance to oral mucosal lesions or potentially malignant disorders, absence of an incarcerated population, or inappropriate study design.

Ultimately, 5 studies met the inclusion criteria and were included in the qualitative synthesis.

All included studies had a cross-sectional design and were conducted in diverse geographical settings, including Europe (Portugal), South America (Brazil), and Asia (India). The majority of studies focused on male prison populations, while one study evaluated female inmates. Sample sizes varied considerably across studies.

The outcomes assessed were heterogeneous and included oral mucosal lesions, potentially malignant oral disorders, oral HPV infection, and general oral health conditions. Some studies specifically reported the prevalence of premalignant lesions, while others provided broader data on oral manifestations from which relevant information on OPMDs could be extracted. A total of five studies were assessed using the adapted Newcastle–Ottawa Scale. Overall, the methodological quality was moderate, with total scores ranging from 4 to 6 out of a maximum of 9 points (Table 1).

Premalignant lesions were reported in up to 34.28% of inmates, with oral submucous fibrosis accounting for 25.43% in one study. Additionally, a high prevalence of oral high-risk HPV infection (81.48%) associated with mild dysplastic changes was observed in female inmates. Oral manifestations overall were reported in up to 90.1% of incarcerated individuals.

Behavioral risk factors were consistently identified across studies. Tobacco use emerged as the most prevalent factor, often in combination with alcohol consumption, illicit drug use, or betel quid chewing, and was strongly associated with the presence of oral lesions and potentially malignant conditions.

A detailed summary of the characteristics of the included studies is presented in Table 2.

Only primary observational studies conducted in incarcerated populations and reporting oral mucosal lesions or potentially malignant conditions were included in the qualitative synthesis.

### 3.2. Results of Individual Studies

The included studies consistently reported a high burden of oral diseases among incarcerated populations, with particular emphasis on oral mucosal lesions and potentially malignant disorders (OPMDs), although the extent and type of outcomes varied across studies.

Rawlani et al. [6] reported one of the highest prevalences of premalignant lesions, identifying OPMDs in 34.28% of inmates, with oral submucous fibrosis (OSMF) present in 25.43% of cases. These findings highlight a substantial burden of lesions with malignant potential, particularly in populations with high exposure to tobacco and betel quid.

Arjun et al. [16] identified a range of oral mucosal lesions among psychiatric inmates, including inflammatory and potentially premalignant conditions. The study highlighted the vulnerability of specific subgroups within prison populations, particularly those with psychiatric comorbidities.

Similarly, Chaudhari et al. [17] identified precancerous and cancerous lesions through screening programs in male inmates, confirming the presence of clinically detectable high-risk lesions in correctional settings.

Zonta et al. [18] focused on viral-related risk factors, reporting a high prevalence of oral HPV infection (81.48%), including high-risk genotypes such as HPV-16 and HPV-59, which were associated with mild epithelial dysplasia. These findings suggest an additional oncogenic pathway contributing to oral carcinogenesis in incarcerated female populations.

Relvas et al. [9] reported oral manifestations in 90.1% of inmates, with a strong association with behavioral risk factors, particularly illicit drug use (86.8%) and smoking (93.7%) (*p* < 0.001). Although the study did not exclusively focus on OPMDs, the high prevalence of oral lesions suggests a substantial burden of mucosal alterations requiring further diagnostic assessment.

Overall, despite heterogeneity in study design and outcome reporting, the findings consistently indicate that incarcerated populations present a high prevalence of oral lesions, including clinically relevant OPMDs. The variability in reported prevalence may be attributed to differences in diagnostic criteria, population characteristics, and exposure to risk factors.

## 4. Discussion

This systematic review aimed to evaluate the prevalence of oral mucosal lesions, with particular emphasis on potentially malignant oral disorders (OPMDs), in prison populations. Although the number of studies specifically addressing OPMDs remains limited, the available evidence consistently suggests a clinically relevant burden of premalignant conditions among incarcerated individuals [1,2,3].

The prevalence of OPMDs in prison populations appears to be influenced by the high exposure to behavioral risk factors. Previous literature has demonstrated that lesions such as leukoplakia and oral submucous fibrosis are strongly associated with tobacco consumption and other carcinogenic habits, which are highly prevalent in incarcerated individuals [4,5,6]. In particular, tobacco smoking remains the most significant modifiable risk factor for oral carcinogenesis, with a well-documented dose–response relationship [4].

In addition to traditional risk factors, emerging evidence highlights the role of oncogenic viral infections. Human papillomavirus (HPV), particularly high-risk genotypes such as HPV-16, has been increasingly recognized as a contributing factor in oral carcinogenesis [7]. The coexistence of HPV infection with other exposures, including tobacco and alcohol use, may exert a synergistic effect, further increasing the likelihood of malignant transformation [8].

Substance abuse also plays a crucial role in oral health deterioration. Illicit drug use has been associated with increased prevalence of dental caries, periodontal disease, and mucosal lesions, all of which may contribute to a pro-inflammatory environment conducive to carcinogenesis [9]. In particular, methamphetamine use has been linked to severe oral conditions, including xerostomia and rampant caries, which may indirectly increase susceptibility to mucosal damage and lesion development [10].

Beyond individual behaviors, structural and environmental determinants intrinsic to the prison setting significantly influence oral health outcomes. Limited access to dental care, lack of preventive programs, and a healthcare system often focused on emergency management contribute to delayed diagnosis of oral lesions [11,12]. Consequently, potentially malignant disorders may remain undetected for prolonged periods, increasing the risk of progression to invasive carcinoma.

The high prevalence of untreated oral diseases and poor oral hygiene among inmates further exacerbates this condition. Several studies have reported significantly higher levels of caries experience and periodontal disease in prison populations compared to the general population, reflecting unmet oral healthcare needs [2,13,14].

Psychosocial and systemic factors may also contribute to disease progression. Chronic stress, nutritional deficiencies, and comorbidities frequently observed in incarcerated individuals may impair immune function and tissue repair mechanisms, thereby facilitating the persistence and progression of mucosal lesions [16,17].

### Limitations of the Evidence

Despite these findings, the available evidence remains limited and heterogeneous. Most studies included in this review are cross-sectional, which precludes causal inference and limits the ability to assess the natural history of OPMDs. Additionally, variability in diagnostic criteria and assessment methods reduces comparability across studies [15].

From a clinical and public health perspective, these findings highlight the urgent need for targeted oral health interventions in prison settings. The implementation of systematic screening programs for early detection of OPMDs, combined with preventive strategies such as tobacco cessation and oral health education, may significantly reduce the burden of disease and improve clinical outcomes [4,11].

## 5. Conclusions

This PRISMA-compliant systematic review demonstrates a clinically relevant burden of potentially malignant oral disorders (OPMDs) among incarcerated populations. Despite the limited and heterogeneous nature of the available evidence, consistent patterns can be identified. In particular, the high prevalence of behavioral risk factors—especially tobacco use, frequently combined with alcohol consumption, illicit drug use, or betel quid chewing—appears to be strongly associated with the development of oral mucosal alterations with malignant potential.

In addition, structural factors inherent to correctional settings, including restricted access to routine dental care, absence of systematic screening programs, and delayed diagnosis, likely contribute to the persistence and progression of precancerous lesions. The identification of high-risk human papillomavirus (HPV) infections in selected cohorts further supports the multifactorial etiology of oral carcinogenesis in this population.

However, the overall strength of the evidence remains limited. The predominance of cross-sectional study designs, the lack of standardized diagnostic criteria, and the insufficient control of confounding variables limit the ability to establish causal relationships and to derive precise prevalence estimates.

This review addresses a relevant gap in the literature and indicates that incarcerated individuals represent a high-risk and underserved population requiring targeted preventive and diagnostic strategies.

Several limitations should be acknowledged. The number of included studies was relatively small, and many did not specifically focus on OPMDs but rather on general oral health outcomes, from which relevant data were extracted. All included studies were cross-sectional, precluding assessment of temporal relationships and disease progression. Furthermore, heterogeneity in diagnostic criteria and outcome assessment limits comparability across studies. The overall methodological quality was moderate (NOS scores ranging from 4 to 6), with limited adjustment for key confounders.

Despite these limitations, the available evidence provides meaningful insights into oral health conditions in correctional settings. From a clinical and public health perspective, incarcerated populations should be considered a priority group for oral health interventions, particularly for the early detection of OPMDs. The integration of routine oral examinations, including systematic mucosal assessment, into prison healthcare services may facilitate earlier diagnosis. In parallel, preventive strategies—such as tobacco cessation interventions, oral hygiene promotion, and risk factor modification—are essential to reduce disease burden. 

Future research should prioritize well-designed longitudinal studies to clarify the natural history of OPMDs and to quantify the risk of malignant transformation over time. The standardization of diagnostic criteria and reporting methods is essential to improve comparability and strengthen the evidence base. In addition, future studies should include more diverse populations, particularly female inmates and underrepresented geographical regions, to enhance external validity. Finally, interventional studies are needed to evaluate the effectiveness of screening and preventive strategies in correctional settings.

## Figures and Tables

**Table 1:** Methodological quality assessment of included studies using the Newcastle–Ottawa Scale (NOS).

Total (0–9)	Outcome (0–3)	Comparability (0–2)	Selection (0–4)	Study
6	2	1	3	Arjun et al. (2014) [16]
6	2	1	3	Relvas et al. (2025) [9]
5	2	1	2	Zonta et al. (2012) [18]
4	2	0	2	Rawlani (2019) [6]
6	2	1	3	Chaudhari et al. (2013) [17]

**Table 2:** Characteristics of included studies.

Study	Design	Population	Location	Outcomes Measured	Key Findings	Main Risk Factors
Relvas et al. (2025) [9]	Cross-sectional	Male inmates	Portugal	Oral lesions, caries	Oral manifestations 90.1%	Drugs, smoking
Zonta et al. (2012) [18]	Cross-sectional	Female inmates	Brazil	HPV, dysplasia	HPV 81.48%	HPV, smoking
Arjun et al. (2014 [16]	Cross-sectional	Psychiatric inmates	India	Oral mucosal lesions	Detected lesions	Tobacco
Rawlani et al. (2019) [6]	Cross-sectional	Inmates	India	Premalignant lesions	34.28% premalignant lesions	Tobacco, betel
Chaudhari et al. (2013) [17]	Cross-sectional	Male inmates	India	Precancer	Detected lesions	Tobacco

## Data Availability

No new data were created or analyzed in this study. Data sharing is not applicable to this article as all data extracted and analyzed are from previously published studies that are cited in the reference list.

## Supplementary Materials

The following supporting information can be downloaded at: https://www.mdpi.com/article/10.3390/dj14050302/s1, Table S1: PRISMA 2020 Checklist for this systematic review.
